# Predicting postoperative pain in children: an observational study using the pain threshold Index

**DOI:** 10.3389/fped.2024.1398182

**Published:** 2024-07-18

**Authors:** Zenghui Liang, Yanle Xie, Shuhan Chen, Jing Liu, Huimin Lv, Bertrand-Geoffrey Muhoza, Fei Xing, Yuanyuan Mao, Xin Wei, Na Xing, Jianjun Yang, Zhongyu Wang, Jingjing Yuan

**Affiliations:** ^1^ Department of Anesthesiology, Pain and Perioperative Medicine, the First Affiliated Hospital of Zhengzhou University, Zhengzhou, Henan, China; ^2^ Cognition and Emotion, Henan Province International Joint Laboratory of Pain, Zhengzhou, Henan, China

**Keywords:** children, perioperative analgesia monitoring, pain threshold index, wavelet index, pain

## Abstract

**Objective:**

While the pain threshold index (PTI) holds potential as a tool for monitoring analgesia-pain equilibrium, its precision in forecasting postoperative pain in children remains unconfirmed. This study's primary aim was to assess the PTI's predictive precision for postoperative pain.

**Methods:**

Children (aged 2–16 years) undergoing general surgery under general anesthesia were included. Within 5 min prior to the patient's emergence from surgery, data including PTI, wavelet index (WLI), heart rates (HR) and mean arterial pressure (MAP) were collected. Subsequently, a 15-min pain assessment was conducted following the patient's awakening. The accuracy of these indicators in discerning between mild and moderate to severe postoperative pain was evaluated through receiver operating characteristic (ROC) analysis.

**Results:**

The analysis encompassed data from 90 children. ROC analysis showed that PTI was slightly better than HR, MAP and WLI in predicting postoperative pain, but its predictive value was limited. The area under the curve (AUC) was 0.659 [0.537∼0.780] and the optimal threshold was 65[64–67]. Sensitivity and specificity were determined at 0.90 and 0.50, respectively. In a multivariable logistic regression model, a higher predictive accuracy was found for a multivariable predictor combining PTI values with gender, BMI, HR and MAP (AUC, 0.768; 95%CI, 0.669–0.866). Upon further scrutinizing the age groups, PTI's AUC was 0.796 for children aged 9–16, 0.656 for those aged 4–8, and 0.601 for younger individuals.

**Conclusions:**

PTI, when used alone, lacks acceptable accuracy in predicting postoperative pain in children aged 2 to 16 years. However, when combined with other factors, it shows improved predictive accuracy. Notably, PTI appears to be more accurate in older children.

## 1 Introduction

Severe postoperative pain in children yields noteworthy enduring consequences and has the potential to induce hyperalgesia and persistent pain (1, 2). A study has shown a median incidence of up to 20% for chronic postsurgical pain (CPSP) in children (3). Effective management of postoperative pain not only alleviates patient discomfort but also enhances satisfaction, reduces postoperative mortality, expedites recovery, and shortens hospital stay duration (4). Therefore, the accurate and timely recognition and treatment of postoperative pain are crucial determinants of patient prognosis.

The Joint Commission on Accreditation of Health Care Organization (JCAHO) designates pain as the fifth vital sign following heart rate, blood pressure, pulse, and breathing (5). Pain assessment serves as an initial stage in pain management, focusing not solely on pain scores but also on effective treatment. Selecting an appropriate pain assessment scale, tailored to the patient's age, level of consciousness, condition, and pain characteristics, is crucial for successful pain management (6). Nonetheless, inadequate assessment frequently hampers pain management, particularly when dealing with patients facing cognitive impairments, which can complicate both pain assessment and treatment (7).

Various physiological parameters, including alterations in the autonomic nervous system, biopotentials, neuroimaging data, biomarkers, and composite algorithms, offer potential avenues for the objective assessment of pain and nociception. While each method shows some promise, robust clinical evidence supporting the the widespread adoption of any specific physiological marker as an objective measure for pain and nociception remains limited (8). The Pain Threshold Index (PTI) is an analytical tool that utilizes a wavelet algorithm to analyze EEG signals from both hemispheres of the forehead, assessing the cerebral cortex's capacity to endure pain stimuli once consciousness fades. Ranging from 0 to 100, this comprehensive EEG parameter was developed by Beijing Easymonitor Technology Co., Ltd. (Beijing, China). A specialized pediatric model facilitates its application in monitoring children throughout the perioperative period. PTI proves valuable for monitoring nociceptive stimuli during general anesthesia among children (9) and for predicting postoperative pain in adults (10). Nevertheless, its ability to predict postoperative pain in children requires further validation.

The primary aim of this study was to investigate the precision of PTI in forecasting the extent of postoperative pain and to ascertain its optimal threshold for discerning moderate to severe postoperative pain in children.

## 2 Materials and methods

### 2.1 Research ethics

This observational study has been approved by the Ethics Committee of the First Affiliated Hospital of Zhengzhou University and registered with the China Clinical Trials Registry (ChiCTR2300073960). Participants in this observational trial obtained the written informed consent of their parents.

### 2.2 Inclusion and exclusion criteria

Children aged 2–16 years, categorized as American Society of Anesthesiologists (ASA) grade I-II who underwent elective general surgery under general anesthesia between February 2023 and June 2023 were included. Exclusion criteria included age less than 2 years or greater than 16 years, pre-existing brain disorders (such as epilepsy, autism, cognitive dysfunction), autonomic nervous system disorders, hepatic and renal diseases, endocrine diseases, arrhythmias, a history of chronic pain before surgery, recent acute upper respiratory tract infection (within the last two weeks), and the use of anticholinergics, vasoactives or esketamine before and during the study period.

### 2.3 Study protocol

Anesthesia induction for all patients consisted of propofol, fentanyl, and cisatracurium, whereas maintenance involved either remifentanil, sevoflurane, or propofol, tailored to the clinical context and anesthesiologist's preference. The anesthesia induction and maintenance protocols could be adapted based on individual clinical needs and anesthesiologist discretion. Subsequent to anesthesia induction, each child underwent PTI and wavelet index (WLI) monitoring in addition to routine anesthesia oversight. WLI, also developed by Beijing Easymonitor Technology Co., Ltd., primarily serves to gauge the depth of perioperative sedation within a range of 0 to100. While comparable to the bispectral index (BIS), the WLI has demonstrated potential superiority over BIS (11). During surgery, the WLI is maintained between 40 and 65, whereas PTI does not entail specific predefined targets.

Following the conclusion of surgery, marked by the completion of skin sutures, patient data were collected within a span of 5 min, prior to the attainment of WLI values exceeding 65. Within this timeframe, PTI, WLI, heart rate (HR), and mean arterial pressure (MAP) readings were recorded. PTI, WLI, and HR were documented every minute, whereas MAP was logged every 2.5 min. Upon removal of the endotracheal tube or laryngeal mask, and upon the patient regaining consciousness in the post anesthesia care unit (PACU), postoperative pain assessments were conducted every 5 min, with pain scores documented within a 15-mine window. Pain evaluation employed varying scales: the FLACC (Face, Legs, Activity, Cry, and Consolability) score for children aged 2–3 years, the Revised Facial Pain Scale (FPS-R) for those aged 4–8 years, and the numerical rating scale (NRS) for individuals aged 9 to 16 years. When a patient exhibited moderate to severe pain (characterized by a pain score exceeding 3) in the PACU, opioid analgesics were administered to provide remedial relief.

### 2.4 Primary and secondary endpoints

The main objective of this study is to evaluate the ability of the average PTI value within 5 min prior to awakening after the procedure to predict the extent of postoperative pain and establish an optimal threshold for distinguishing between mild and moderate to severe pain. Supplemental goals include exploring the ability of PTI and WLI values to predict postoperative pain in relation to age. Additionally, the investigation aims to analyze the correlation between these values and the highest postoperative pain score observed in the post anesthesia care unit (PACU).

### 2.5 Sample size calculation

We calculated the sample size based on the AUC value. Based on the prior literature, the area under the curve (AUC) for PTI in predicting moderate to severe pain post-surgery was reported as 0.77[10]. The null hypothesis (H0) considered an AUC of 0.5, while the alternative hypothesis (H1) assumed an AUC = 0.77. With a significance level (*α*) of 0.05 and a power (1-β) of 0.9, the ratio of patients experiencing mild and moderate to severe pain after surgery was 1:1. The sample size estimation was performed by PASS15.0, indicating a requirement of at least 44. Factoring in a projected dropout rate of 20%, a total of 56 patients were deemed necessary. Ultimately, 90 patients were included in the study for comprehensive statistical analysis.

### 2.6 Statistical analysis

Continuous variables were presented as either mean ± standard deviation (SD) or median with the interquartile range (25th and 75th percentile), while categorical variables were reported as frequencies and percentages. The predictive accuracy of PTI, WLI, HR, and MAP for postoperative pain severity was assessed through receiver operating characteristic (ROC) analysis. The optimal cut-off value was defined as the point with the highest sum of sensitivity and specificity and the confidence interval for the optimal cutoff of was assessed by bootstrapping with 1,000 repetitions. Spearman's correlation test was employed to examine the relationship between these indices and postoperative pain scores. Multivariate logistic regression analysis was used to evaluate the correlation between different independent variables and postoperative moderate to severe pain. Outcome probabilities estimated from multivariate logistic regression models were used as a predictor for ROC analysis. The *P*-value was considered statistically significant at <0.05. Statistical analyses were conducted with SPSS 25.0 software (SPSS, Inc., Chicago, 150 IL, USA) and R Software (V4.3.1; R Core Team 2023) via the pROC R package (V1.18.5; Robin and Turck 2011).

## 3 Results

### 3.1 Study population

A total of 90 patients were enrolled in the study and subjected to comprehensive statistical analysis, comprising 31 individuals aged 2–3 years, 44 aged 4–8 years, and 15 aged 9–16 years. Detailed clinical characteristics were described in Table 1. During the 15 min assessment in the post anesthesia care unit (PACU), the maximum pain score per patient was categorized as either mild (pain score ≤ 3; *n* = 40) or moderate to severe (pain score > 3; *n* = 50).

**Table 1:** Cohort demographic characteristics and perioperative data overall and grouped by age.

	Overall (*n* = 90)	2–3 (*n* = 31)	4–8 (*n* = 44)	9–16 (*n* = 15)
Age (years)	4.0 (3.0–7.0)	3.0 (2.0–3.0)	5.0 (4.0–6.8)	11.0 (10.0–12.0)
Gender (Male/Female, *n*)	74/16	28/3	34/10	12/3
Height (cm)	113.1±23.8	92.5±8.3	115.0±16.3	150.4±13.6
Weight (Kg)	19.5 (15.0–29.0)	14.4 (13.0–16.0)	21.0 (17.3–27.5)	36.0 (33.0–45.0)
ASA physical status (I/II)	69/21	24/7	34/10	11/4
Duration of surgery (min)	70.5 (45.0–100.0)	74.0 (27.0–89.0)	56.0 (27.5–72.0)	69.0 (28.0–110.0)
Highest pain score (*n*)	0–3: 40	0–3: 13	0–3: 21	0–3: 6
	4–6: 50	4–6: 18	4–6: 23	4–6: 9
Type of surgery				
Urological surgery (*n*)	53	23	23	7
General surgery (*n*)	37	8	21	8

Data are expressed as the mean ± SD, median (interquartile range), or *n*; ASA: American society of anesthesiologists.

### 3.2 Predictive ability of PTI, WLI, HR, and MAP

**Figure 1:**
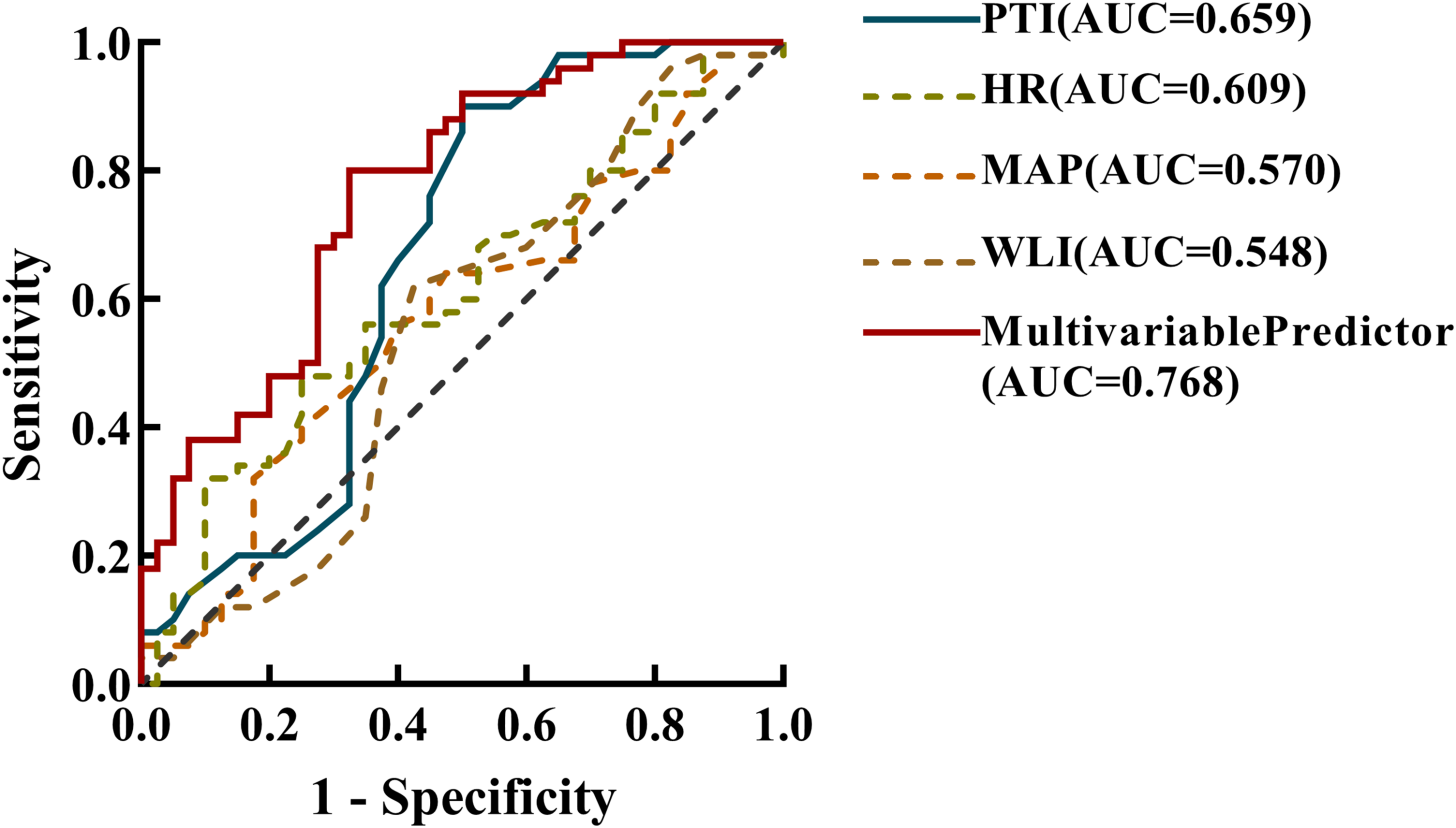
Receiver operating characteristic (ROC) analysis, incorporating the area under the curve (AUC) was employed to analyze the prediction accuracy for the mean pain threshold index(PTI), wavelet index(WLI), heart rate(HR), and mean arterial pressure (MAP) within the 5 min interval preceding emergence from general anaesthesia post-surgery, as well as the multivariate prediction model. This analysis aimed to differentiate between mild (0–3) and moderate-to-severe (4–10) levels of postoperative acute pain. The estimated multivariable predictor of the final model contained mean PTI, HR, MAP values within the last 5 min before emergence from general anaesthesia, as well as gender and BMI.

The mean values of PTI, WLI, HR, and MAP were subjected to ROC analysis alongside the highest pain score recorded in the PACU following surgery. The outcomes revealed that PTI (AUC, 0.659; 0.537–0.780; *P *= 0.010) exhibited slightly superior predictive accuracy compared to HR (AUC, 0.609; 95% CI, 0.492–0.726; *P *= 0.077) and MAP (AUC, 0.570; 95% CI,0.450–0.690; *P *= 0.254). Conversely, WLI (AUC, 0.548; 95% CI, 0.424–0.673; *P *= 0.433) was less effective at predicting postoperative pain than HR and MAP (Figure 1).

Logistic regression analysis showed that gender, Body Mass Index (BMI), PTI, HR, and MAP were associated with immediate postoperative pain. The OR showed that women were 5.5-fold more likely to experience moderate to severe pain after surgery than men. For every 1 increase in BMI, the odd of moderate to severe postoperative pain is reduced by 25%. In addition, for every 1 increase in PTI, HR and MAP, the odds of moderate to severe postoperative pain increased by 1.71, 1.04 and 1.08 fold, respectively (Table 2). It was found that the multivariate prediction model had the highest predictive power for postoperative pain (AUC, 0.768; 95% CI, 0.669–0.866) (Figure 1). Sensitivity analysis by constructing a reduced model excluding the PTI variable showed a significantly lower AUC (0.675; 95% CI, 0.558–0.792) compared to the full model incorporating PTI (AUC, 0.768; 95% CI, 0.669–0.866; *P *= 0.035, DeLong test), indicating that PTI significantly improves the model's discriminative ability and is a crucial predictor of immediate postoperative pain.

**Table 2:** Multivariate logistic regression analysis of influencing factors of moderate to severe pain in children aged 2–16 years after surgery.

Independent variable	*β*	SE	Wald	*P*	OR	95%CI	
						Lower	Upper
Gender	1.712	0.849	4.071	0.044	5.540	1.050	29.228
Age	0.161	0.110	2.165	0.141	1.175	0.948	1.457
BMI	−0.283	0.126	5.030	0.025	0.754	0.588	0.965
ASA	−1.459	0.788	3.427	0.064	0.232	0.050	1.089
Type of surgery	0.610	0.638	0.913	0.339	1.840	0.527	6.423
Intraoperative dose of remifentanil (*μ*g/kg/min)	0.427	0.328	0.110	0.921	1.533	0.000	7.935
Duration of surgery (min)	−0.004	0.008	0.242	0.623	0.996	0.981	1.011
PTI	0.535	0.186	8.303	0.004	1.708	1.187	2.458
WLI	0.181	0.404	0.201	0.654	1.198	0.543	2.643
HR	0.041	0.019	4.380	0.036	1.042	1.003	1.082
MAP	0.073	0.034	4.520	0.034	1.075	1.006	1.150

BMI, body mass index; ASA, American Society of Anesthesiologists; PTI, pain threshold index; WLI, wavelet index; HR, heart rates; MAP, mean arterial pressure.

**Figure 2:**
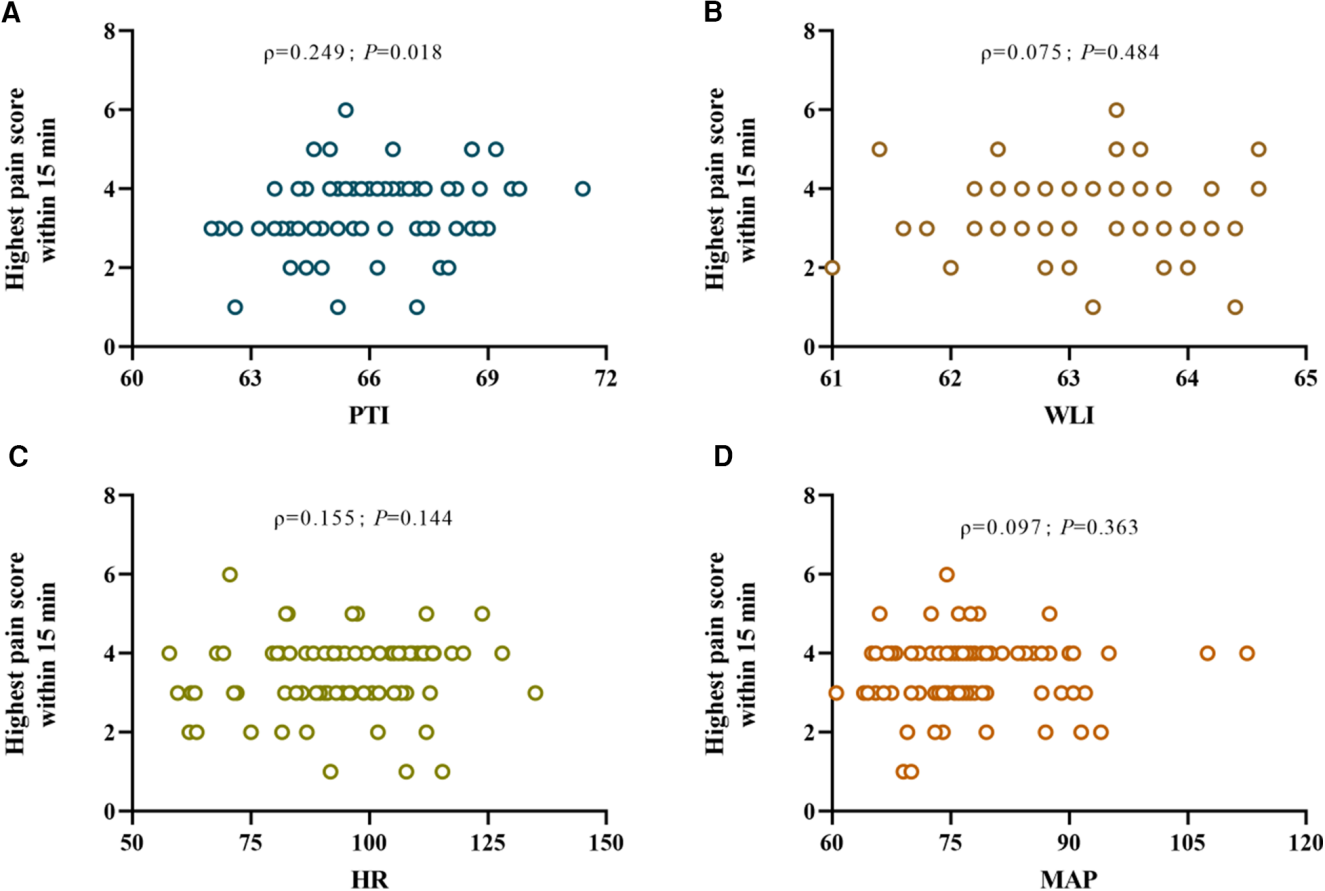
Scatter plots (**A**), (**B**), (**C**), and (**D**) depict the correlations between the mean pain threshold index (PTI), wavelet index (WLI), heart rate (HR), and mean arterial pressure (MAP) respectively within the 5 minute interval prior to emergence from general anesthesia after surgery and the highest postoperative pain ratings observed upon awakening, where ρ denotes Spearman's rank correlation coefficient for the correlation between each respective nociception index and postoperative pain levels.

The mean values of PTI, WLI, HR and MAP before the patient's awakening post-surgery were subjected to Spearman correlation analysis in relation to the highest pain score recorded in the PACU. The results showed that only PTI exhibited an association with postoperative pain scores (*ρ *= 0.249, *P *= 0.018). Conversely, WLI (*ρ *= 0.075, *P *= 0.484), HR (*ρ *= 0.155, *P *= 0.144), and MAP (*ρ *= 0.097, *P *= 0.363) displayed no statistically significant association with postoperative pain scores (Figure 2).

The optimal cut-off for PTI to predict postoperative pain in children aged 2–16 years was 65, with a sensitivity of 0.90 and a specificity of 0.50. Upon conducting ROC analysis across three distinct age categories, it was evident that PTI and WLI displayed greater predictive precision for postoperative pain in older children compared to their younger counterparts. Among children aged 9–16 years, the AUC for PTI stood at 0.796, and for WLI, it reached 0.620. In comparison, for children aged 2–3 years, the respective AUCs were 0.656 for PTI and 0.526 for WLI. Similarly, for children aged 4–8 years, the AUCs were 0.601 for PTI and 0.608 for WLI (Table 3).

**Table 3:** The area under the receiver operating curve (ROCAUC) of pain threshold index (PTI) and wavelet index (WLI) across different age groups to distinguish between mild and moderate to severe pain.

Age group (year)	ROC AUC (95% confidence interval)	Cut off (95% confidence interval)	Sensitivity	Specificity
2–16	PTI 0.659 (0.537–0.780)	65 (64–67)	0.90	0.50
	WLI 0.548 (0.424–0.673)	63 (62–64)	0.62	0.58
2–3	PTI 0.656 (0.439–0.873)	65 (64–68)	0.94	0.54
	WLI 0.526 (0.306–0.746)	62 (62–64)	0.89	0.31
4–8	PTI 0.601 (0.424–0.779)	65 (64–68)	0.83	0.52
	WLI 0.608 (0.432–0.783)	63 (62–64)	0.74	0.62
9–16	PTI 0.796 (0.536–1.000)	64 (62–64)	1.00	0.67
	WLI 0.620 (0.311–0.930)	63 (62–64)	1.00	0.33

Optimal cut-off values, corresponding sensitivity and specificity were calculated.

## 4 Discussion

“Nociception” refers to the neural processes of encoding and processing noxious stimuli (12). Nociception is challenging to quantify in anesthetized or unconscious patients. Essentially, subcortical areas are in charge of integrating nociception (13). In pediatric cases, postoperative pain often goes unnoticed due to the patient's age. As the focus on perioperative analgesia monitoring intensifies, a range of pain assessment tools have been developed. These tools include skin conductance (SC) (14), analgesia and nociception index (ANI) (15–17), and surgical stress index (SPI) (18), which can be utilized to predict postoperative pain severity in children. However, these methods possess certain limitations and are influenced by factors like anticholinergics, vasoactive drugs, the patient's level of consciousness, and emotional state. The search for specific, objective indicators that reflect the balance between analgesia and pain persists.

As established by numerous studies, the presence of pain is capable of inducing substantial alterations in electroencephalogram (EEG) signals, thereby suggesting that EEG monitoring serves as a reliable indicator of pain perception (13, 19–21). In recent years, the Pain Threshold Index (PTI) has emerged as a novel tool for monitoring pain. It captures conductive signals from both hemispheres of the cerebral cortex and employs wavelet algorithms to analyze EEG data across multiple dimensions, rendering it advantageous in several aspects (10).

This study aimed to assess predictive indicators for postoperative pain in children, with a focus on the Pain Threshold Index (PTI). While the sensitivity analysis identified PTI as an independent predictor of postoperative pain, the study showed that PTI alone yielded suboptimal diagnostic accuracy for postoperative pain in children aged 2–16. Although PTI showed a slight advantage over heart rate (HR), mean arterial pressure (MAP), and wavelet index (WLI), its area under the ROC curve (AUC) was only 0.659, falling below the generally accepted standard of 0.7. In multivariate logistic regression models, acceptable diagnostic accuracy can be observed when PTI values are combined with gender, BMI, HR, and MAP. The results of Spearman's rank correlation analysis revealed that only PTI was associated with postoperative pain scores, but the correlation was weak and basically had no clinical value. These results suggest that the use of PTI alone in identifying moderate to severe pain postoperatively in children aged 2–16 years has limited value. Therefore, the primary conclusion of this study is that, when used in isolation, PTI lacks acceptable accuracy in predicting postoperative pain in children aged 2–16. However, upon delving into age-related variations, it became apparent that both PTI and WLI demonstrated greater effectiveness when employed in older children (9–16 years), showcasing heightened predictive precision compared to younger counterparts (2–8 years).

Nevertheless, in a multivariate logistic regression model combining PTI with factors such as gender, BMI, HR, and MAP, the predictive accuracy for postoperative pain improved, with an AUC of 0.768, demonstrating better predictive value. This suggests that the combined use of multiple physiological indicators may enhance the accuracy of predicting postoperative pain in children in clinical practice. Particularly, factors such as gender, BMI, HR, and MAP were found to be associated with postoperative pain in multivariate analysis, underscoring the importance of considering patient physiological characteristics in predicting pediatric postoperative pain.

In a study of PTI in predicting postoperative pain in adults, PTI demonstrated an optimal cut-off value of 53, effectively distinguishing between mild and moderate to severe postoperative pain. Sensitivity and specificity were recorded at 62.50% and 90.91%, respectively. Remarkably, the predictive accuracy of PTI outperformed that of the Surgical Stress Index (SPI) (10). This study uncovered an optimal cut-off value of 65 for PTI, effectively distinguishing between mild and moderate to severe pain in children aged 2–16 years. Sensitivity and specificity stood at 90% and 50%, respectively. EEG patterns in children exhibit age-related variations, with distinct EEG characteristics at different developmental stages. As the brain matures, EEG patterns in older children resemble those of adults (22, 23). Notably, the variations in PTI values between mild and moderate to severe pain after surgery in adults and children could be attributed, in part, to differences in EEG characteristics (24). Furthermore, A study has shown that PTI-guided analgesia during gynecological operations in adults resulted in a 25% reduction in remedial analgesia (25), suggesting that we could further investigate the clinical efficacy of PTI in the pediatric population by employing different thresholds and more appropriate protocols.

WLI serves as an indicator reflecting the level of sedation during general anesthesia, with an appropriate depth of sedation typically maintained between 40 and 60 throughout the surgery (26, 27). During the study, we found that sustaining the WLI below 60 necessitated a higher anesthetic dose than the standard administration, potentially leading to reduced blood pressure and a slower heart rate. A comparable trend was evident with the use of BIS. In scenarios where volatile anesthetics are adjusted to maintain specific Minimum Alveolar Concentration (MAC) values, BIS values tend to decline as the age of children increases (28). Additional research indicates that maintaining the BIS at 50 prompts a notably greater requirement for propofol in children as compared to adults (29). These findings collectively underscore the necessity for cautious consideration when employing EEG-derived markers such as PTI and WLI, especially in the context of younger children.

In this research, further analysis of age groups revealed that PTI performed better in predicting postoperative pain in children aged 9–16 (AUC of 0.796) compared to those aged 2–8, possibly due to age-related variations in pediatric EEG patterns that differ from adults. This highlights the need for caution in applying PTI and similar physiological indicators in children, requiring different analyses and standards based on age groups.

Additionally, Spearman correlation analysis between PTI and postoperative pain scores indicated a weak but significant correlation. This suggests that while PTI is moderately associated with postoperative pain, its predictive value is limited when used in isolation. In practical applications, a comprehensive assessment of pediatric postoperative pain may require the integration of other physiological parameters and clinical information.

Several limitations were present in this study. The employment of *post hoc* subgroup analysis, involving 15 patients in the 9–16 year old group, 31 patients in the 2–3 year old group, and 44 patients in the 4–8 year old group, led to uneven representation across age groups. This uneven distribution, particularly notable in the 9–16 year old group, might influence the precision of PTI prediction within this age bracket. Larger multicenter observational studies may improve the interpretation of this research. Second, distinct age groups were subjected to varied pain assessment scales, each with its own scoring criteria, potentially impacting the classification of pain severity. Third, we did not record the crucial variable of opioid drugs during the observation, and we will include this variable in subsequent studies. Finally, it should be acknowledged that the AUC value of the multivariable regression model is calculated based on in-sample predicted values, rather than cross-validated values. Consequently, the AUC value may be slightly biased upwards, as the model might be somewhat overfit to the current sample. Future studies should employ cross-validation or other techniques to more accurately assess the model's performance and mitigate the risk of overfitting.

## 5 Conclusion

In conclusion, PTI was slightly better than HR and MAP in predicting postoperative pain in children aged 2 to 16 years, but appeared to lack acceptable diagnostic accuracy. PTI used in older children had a high diagnostic accuracy. Future studies with larger sample sizes are needed to verify its clinical value in older children. Further more, in-depth analyses of other physiological parameters and clinical features may contribute to the development of more accurate predictive models.

## Data Availability

The original contributions presented in the study are included in the article, further inquiries can be directed to the corresponding authors.
